# Public Health Value of a Hypothetical Pneumococcal Conjugate Vaccine (PCV) Introduction: A Case Study

**DOI:** 10.3390/vaccines10060950

**Published:** 2022-06-15

**Authors:** Nathorn Chaiyakunapruk, Dayoung Song, Julia Lynch, Jerome H. Kim, Piyameth Dilokthornsakul, Tawee Chotpitayasunondh, Vittal Mogasale

**Affiliations:** 1 Department of Pharmacotherapy, University of Utah College of Pharmacy, Salt Lake City, UT 84112, USA; 2 IDEAS Center, Veterans Affairs Salt Lake City Healthcare System, Salt Lake City, UT 84112, USA; 3 Policy and Economic Research Department, International Vaccine Institute, SNU Research Park, 1 Gwanak-ro, Gwanak-gu, Seoul 8826, Korea; dysong0211@gmail.com; 4 International Vaccine Institute, SNU Research Park, 1 Gwanak-ro, Gwanak-gu, Seoul 8826, Korea; julia.lynch@ivi.int (J.L.); jerome.kim@ivi.int (J.H.K.); 5 Center for Medical and Health Technology Assessment (CM-HTA), Department of Pharmaceutical Care, Faculty of Pharmacy, Chiang Mai University, Chiang Mai 50210, Thailand; piyameth.dilok@cmu.ac.th; 6 Queen Sirikit National Institute of Child Health (Children’s Hospital), Bangkok 10400, Thailand; cctawee@gmail.com

**Keywords:** VALUE of vaccine assessment, cost-effectiveness analysis, pneumococcal conjugate vaccines (PCV), vaccine introduction

## Abstract

Background: Understanding the public health value of a vaccine at an early stage of development helps in valuing and prioritizing the investment needed. Here we present the potential cost-effectiveness of an upcoming 12 valent pneumococcal conjugate vaccine (PCV 12) in the case study country, Thailand. Methods: The cost-effectiveness analysis included a hypothetical scenario of three doses (2 + 1 regimen) PCV12 introduction in the national immunization program of Thailand compared to no PCV, PCV10, and PCV13 among <6 months old from a societal perspective with a lifetime horizon and one-year cycle length. Data from Thailand, as well as assumptions supported by the literature, were used in the analysis. The price of PCV12 was assumed similar to that of PCV10 or PCV13 for GAVI’s eligible countries based on inputs from stakeholder meeting. A one-way sensitivity analysis was conducted using 0.5–1.5 times the base price of PCV12. Results were presented in incremental cost-effectiveness ratio (ICER) in terms of monetary value per quality-adjusted life-year (QALY) gained. Results: Vaccination with PCV12 among a hypothetical cohort of 100,000 Thai children is expected to avert a total of 5358 cases which includes 5 pneumococcal meningitis, 43 pneumococcal bacteremia, 5144 all-cause pneumonia, and 166 all-cause acute otitis media compared to no vaccination. The national PCV12 vaccination program is a cost-saving strategy compared to the other three strategies. The one-way sensitivity analysis showed PCV12 is a cost-saving strategy when 1.5 times the base price of PCV12 was assumed. Conclusions: Within the limitations of hypothetical assumptions and price points incorporated, the study indicates the potential public health value of PCV12 in Thailand.

## 1. Introduction

Vaccines are innovative life-saving tools against infectious diseases and have contributed to reducing childhood mortality. In advanced economies such as the United States of America, a 95% reduction in 9 vaccine-preventable diseases is attributed to vaccines [1]. Life-threatening infectious diseases, such as smallpox, have been eradicated, and polio is on the verge of eradication as a result of successful vaccination programs [2]. As the number of vaccines introductions targeting specific diseases increases, the total cost of a vaccination package per child has increased steeply, especially in low and middle-income countries (LMICs) [3]. Innovations to reduce the cost of individual vaccines within the immunization basket are a constant global health need.

The research and development that leads to innovations often need significant financial investments from vaccine developers. In recent years, many philanthropic donors, nonprofit organizations, and vaccine developers, often referred to as product development partnerships have collaborated to create low-cost vaccines [4,5]. Such monetary investments need both financial and public health justification. The World Health Organization (WHO) and other global partners have recently highlighted the need for such justifications from a multi-stakeholder perspective categorized into three groups. First, vaccine manufacturers need financial justification for investment in vaccine development and innovations that can be quantified through return on investments, often referred to as a business case. Second, from their public health perspective, global health agencies traditionally look for health benefits that can be achieved through vaccine introductions. This information is generated through vaccine introduction case studies presenting findings from an economic evaluation [6,7]. Typically, vaccine introduction case studies are done after the late stage of vaccine development; however, more recently, conducting such evaluations in the early stage is becoming common. Third, there has been increased awareness of the broader socio-economic benefits of vaccination in recent years, which has led the global health community to develop research approaches that can measure this additional benefit of vaccination [8,9]. Recognizing all these factors, WHO and other global health partners have combined these three elements under one framework designated the Full Value of Vaccine Assessment [10].

This paper presents a case study of the Public Health Value of innovation in a pneumococcal conjugate vaccine (PCV) still under development. As this is an introduction case study, the paper describes the background information on PCV and innovations, methods of selecting a country for a case study, and economic evaluation of introducing an innovative PCV product in the chosen country to understand its public health value.

## 2. Method

### 2.1. PCVs Pneumococcal Conjugate Vaccines and Innovation

Pneumonia is the leading cause of death for children under five in developing countries [11,12]. According to WHO, among 5.83 million deaths of children < 5 years of age in 2015, 294,000 were estimated to be caused by pneumococcal infections [13]. Most of the child deaths related to pneumonia occur in developing countries, with the most significant incidence occurring in sub-Saharan Africa and South Asia [14,15]. Currently, 6–11 serotypes account for ≥70% of all invasive pneumococcal disease (IPD), and case fatality rates from IPD in children can be high, ranging up to 20% for septicemia and 50% for meningitis in LMICs [16]. Pneumococcal disease is, therefore, the leading vaccine-preventable cause of death in young children according to WHO [13].

### 2.2. The Overview of Current Pneumococcal Conjugate Vaccines

Vaccines have been used to prevent pneumococcal disease for more than 30 years. Currently, there are two different types of pneumococcal vaccines available on the market: (1) a 23-valent polysaccharide vaccine (PPV23) available since the early 1980s, and (2) conjugate vaccines, including two vaccines available since 2009, a 10-valent (PCV10) produced by GlaxoSmithKline (GSK) and a 13-valent (PCV13) produced by Pfizer (Table 1). The first conjugate vaccine was a 7-valent (PCV7) and is gradually being removed from the market with the expansion of PCV10 and PCV13. Unlike conjugate vaccines, polysaccharide vaccines are associated with poor or absent immunogenicity in children <2 years of age and failure at any age to induce an anamnestic antibody response upon revaccination. It is not recommended for use in infants [17]. The favorable safety profile of the PCV7 (serotypes 4, 6B, 9V, 14, 18C, 19F, and 23F) vaccine is well established, and several studies have shown that PCV10 (serotypes 1, 4, 5, 6B, 7F, 9V, 14, 18C, 19F, and 23F) and PCV13 (serotypes 1, 3, 4, 5, 6A, 6B, 7F, 9V, 14, 18C, 19A, 19F, and 23F) have similar safety profiles to that of PCV7 when administered to infants and young children [17]. Even though a new PCV10 produced by the Serum Institute of India (serotype 1, 5, 6A, 6B, 7F, 9V, 14, 19A, 19F, 23F) has been prequalified by the World Health Organization, it has not been approved and available in Thailand. Thus, it was not listed as a comparator in this study. In addition, a recent review compared publicly available PCV decision-making tools concerning the WHO guidelines for economic evaluations of immunization programs [18].

### 2.3. PCV Global Market Analysis

Gavi, the Vaccine Alliance (Gavi), set an ambitious goal to immunize 300 million people by 2025 and forecasts the estimated demand of 1.1 billion doses of PCV in 2020–2025 [19]. To meet this forecasted demand, the price of the vaccine is a crucial part of supply and implementation. PCV manufacturers have committed to supplying PCV to eligible countries under Advance Market Commitment (AMC) agreement with GAVI, but the price remains relatively high. In 2013, the price of both GSK and Pfizer Inc’s PCV single-dose vial was US $3.40, which was reduced to US $3.05 in 2017 [20]. In 2018, Pfizer committed to supplying PCV for the retail price of US $2.95 for a four-dose vial and US $2.90 in 2019. Though the price of PCV has been reduced over the years, it remains significantly more expensive than vaccines for other infectious diseases. Some studies suggest that, in humanitarian settings, one complete regimen of PCV costs up to 20 times more than measles-containing vaccines [21]. Therefore, introducing high-priced vaccines such as PCV in low and middle-income countries, particularly those that are not eligible for Gavi, imposes a financial burden on families and the government and limits the accessibility to those in need. Thus, there is a growing need for innovations and technology that lower the cost of vaccines, along with strategic market supply incentives and competition to meet the projected 1.1 billion dose demand for PCV by 2025 [22].

### 2.4. Introduction of PCV12

SK bioscience, a Korean vaccine manufacturer, is developing an affordable 12 valent pneumococcal conjugate vaccine known as SK-PCV12, which contains all but one (serotype 3) of the serotypes represented in PCV13 (Table 2). The goal is to reduce mortality and morbidity by preventing pneumococcal disease in developing countries through low-cost vaccines targeted at LMICs. Bill and Melinda Gates Foundation supported this vaccine development effort. Table 2 below shows the target product profile of SK-PCV12, which is still in development. A country case study was conducted to project evidence and understand the potential health benefits of PCV12 introduction that can inform the value of vaccine development.

## 3. Country Selection

### 3.1. Selection of Country for Case Study

**Figure 1:**
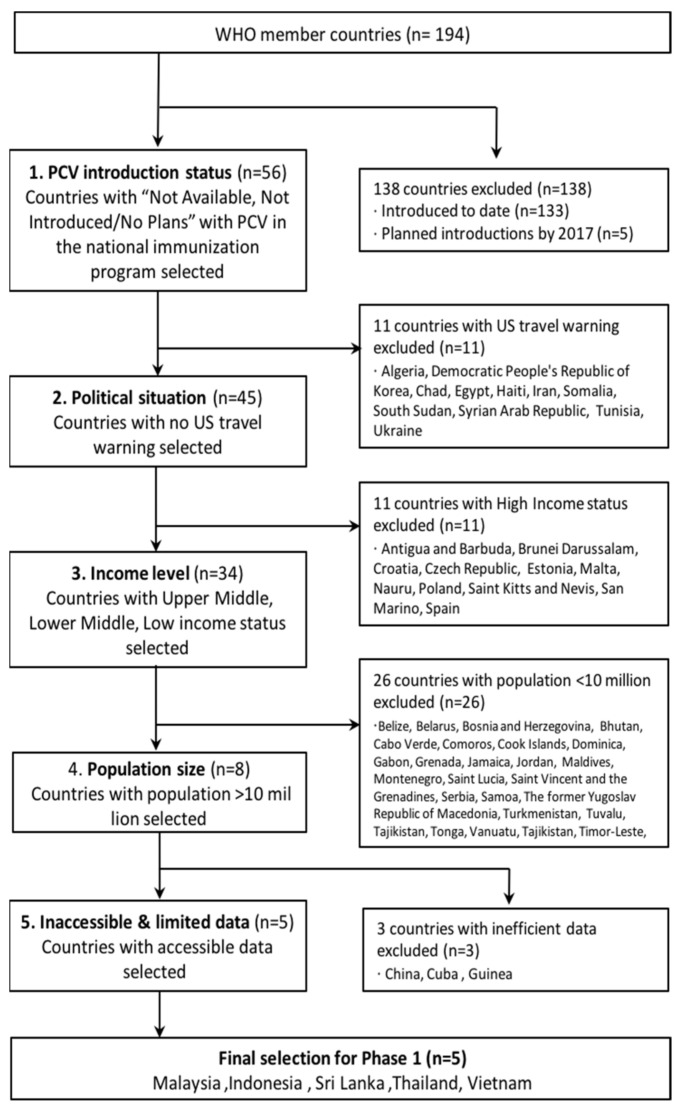
Flowchart of the selection process of Phase.

To introduce PCV into high-risk populations, the International Vaccine Institute (IVI) developed a framework to shortlist target countries. This framework aimed to forecast the “potential demand (estimated hypothetical future demand)” for PCV in each country and prioritize those countries according to the demand. The selection process consists of two phases: Phase (1) Shortlisting eligible countries for PCV introduction, and Phase (2) Prioritization of countries by disease burden and in-country capacity to introduce PCV. Because PCV introduction will be targeted to specific high-risk, low and middle-income countries (LMIC), it was essential to analyze the country’s status of PCV programs on the national level, the capability to introduce the vaccine, as well as the size of the population to be targeted for PCV. In Phase 1, the candidate countries were short-listed based on the availability of PCV in the national immunization program, the US travel warning status, low and middle-income country status according to World Bank Gross National Income (GNI) status, the population status of more than 10 million population, and availability of accessible health data (Figure 1). Among United Nations member states, five countries, Malaysia, Indonesia, Sri Lanka, Thailand, and Vietnam were shortlisted based on Phase 1 criteria. Phase 2 focused on comparing the disease burden of each country and in-country capability by reviewing pneumonia disease burden, epidemiological data, and accessibility of surveillance system. A semi-quantitative scoring system was developed to assess the availability of surveillance data and the in-country capacity of each country shortlisted from Phase 1. The survey tool was shared with World Health Organization (WHO) officers and regional experts to obtain their opinions on the country’s capacity to conduct a PCV case study, its perceived usefulness of case study, and its utilization by policymakers in the five shortlisted countries. Thailand, followed by Malaysia, received the highest scores in Phase 2. Based on these findings, Thailand was selected for the final case study representing an LMIC that has not introduced PCV yet but has a high potential to introduce PCV in the future.

### 3.2. Stakeholder Meeting

After the selection process, the PCV introduction case study aimed to determine the cost-effectiveness and budget impact analysis of adopting PCV12 into the National Universal Immunization Program compared to no PCV, PCV10, and PCV13 among children less than six months old for the national vaccination program in Thailand. After the initial analysis, a stakeholder meeting was conducted on 3 April 2018 to present the study methodology, the data, and assumptions to be used in the analysis and initial results. The stakeholders and experts included clinical practitioners, representatives from the medical society, representatives of decision makers/payers/government sector, researchers, representatives of consumers, and other health experts in Thailand. Their involvement was crucial in setting the parameters because they are in-country experts with an understanding of the health policy context of Thailand and the potential users for the country case study. Stakeholder engagement was one of the critical processes in conducting the analysis and making the results useful to the policymakers.

## 4. Case Study

### 4.1. Economic Analysis

A cost-effectiveness analysis was undertaken to estimate costs and health outcomes of PCV12 compared to no PCV, PCV10, and PCV13 among children less than six months old for the national immunization program in Thailand. A hypothetical cohort of 100,000 children was modeled to mimic the clinical consequence of vaccination. The intervention of interest is the national vaccination program for PCV12, 10, 13 with two-dose and one-booster dose (2 + 1 schedule). Because some cases of IPD (invasive pneumococcal disease), including meningitis, have long-term sequelae, i.e., hearing loss, epilepsy, the lifetime time horizon was chosen in this study. The discount rate of 3% is used for both costs and health outcomes [23]. Results were presented as incremental cost-effectiveness ratio (ICER) in terms of monetary value (USD (Thai Baht; THB) per quality-adjusted life-year (QALY) gained. This study was undertaken using a societal perspective as recommended by Thailand’s Health Technology Assessment (HTA) Guideline [24].

### 4.2. Vaccination Impact Estimation

**Figure 2:**
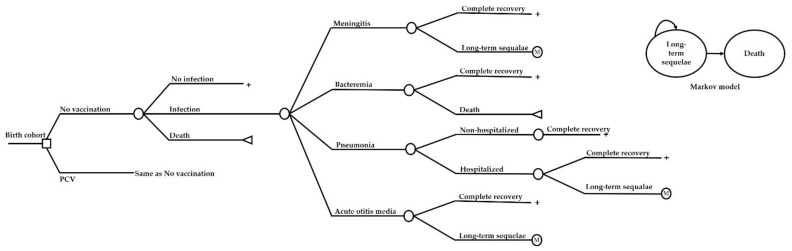
Schematic diagram representing the decision tree for assessing the cost-effectiveness of Pneumococcal vaccination among children. Long-term sequelae were based on the previous infection. Patients with meningitis could have epilepsy, hearing loss, or neurodevelopmental impairment, while those with hospitalized pneumonia could have chronic lung disease. Patients with acute otitis media could have long-tern hearing loss.

A hybrid model consisting of a decision tree and a Markov model was constructed based on the natural history of diseases from pneumococcal infection (Figure 2). The decision tree model captures the incidence of IPD, including meningitis and bacteremia, all-cause pneumonia, all-cause acute otitis media (AOM) during the first five years of life. The reason for choosing 0–5 years is that most pneumococcal infections occur during this time. Subjects with meningitis could have three clinical consequences, including full recovery, recovery with sequelae, and death. Long-term sequelae were based on the type of infection. Patients with meningitis could have epilepsy, hearing loss, or neurodevelopmental impairment, while those with hospitalized pneumonia could have chronic lung disease. Patients with acute otitis media could have long-term hearing loss. The Markov model captured the long-term cost and outcomes of all subjects with sequelae and no sequelae throughout their life. The cycle length of the model was one year. It was assumed that more than pneumococcal infection is possible during the lifetime. However, when individuals had sequelae, they were not to be infected and stayed with the sequelae or death. Model inputs were obtained from various sources, including systematic review and meta-analysis, literature, and an electronic healthcare utilization database obtained from National Health Security Office (NHSO). Vaccine effectiveness (VE) against IPD, all-cause pneumonia and all-cause AOM were estimated based on vaccine efficacy estimates reported in systematic reviews, meta-analyses, and literature. Specifically, vaccine efficacy of PCV12 was assumed to be non-inferior to PCV13 because the prevalence of serotype 3 is very low among children. However, because of no actual evidence of the efficacy of PCV12, we performed sensitivity analyses by lowering vaccine efficacy by 10–30% of PCV13. The 100% vaccine coverage was assumed. The duration of vaccine protection was assumed to be five years. All inputs are shown in Table 3.

### 4.3. Costs and Outcomes

This study was undertaken from the societal perspective; cost data, therefore, included both direct medical and direct nonmedical costs. It was assumed that lost or impaired ability to work or engage in leisure activities due to morbidity would be captured in the disutility of QALY; indirect costs, therefore, were not included, to evade double counting [41]. The price of PCV12 was assumed to be the same as PCV13 purchased for GAVI-eligible countries (3.05 USD/dose). The assumption was based on the proposed price of the vaccine developer, which would like to improve vaccine accessibility at affordable price. The price of PCV10 (16.0 USD/dose) and PCV13 (16.2 USD/dose) were the median prices of each vaccine in the most updated data from the WHO price database (V3P database).[40] The vaccine referent prices were based on the price data of upper-middle-income countries with a similar purchasing volume of 1.8–3.0 million doses/year, similar to the Thailand context [40]. The cost of the vaccination program included vaccine acquisition cost and wastage cost based on the wastage rate from Thailand’s survey study which was approximately 1.1% of the vaccine price [42]. Direct medical costs for hospitalized episodes of meningitis, bacteremia, pneumonia, and nonhospitalized pneumonia and AOM, and annual costs were estimated using the NHSO database (2011–2016) from patients with a primary diagnosis of epilepsy, hearing loss, neurodevelopmental impairment as described above.

### 4.4. Base-Case Analysis

Primary outcomes of interest were the number of pneumococcal infection cases prevented among a hypothetical cohort of children under five years old, incremental costs, life-year gained, QALYs gained, and incremental cost-effectiveness ratio (ICER). For base-case analysis, we calculated the expected lifetime costs and outcomes for all options compared to no vaccination as a reference. The interpretation of the cost-effectiveness of the findings was based on an official willingness-to-pay (WTP) of the Thai Health Economic Working Group (HEWG) threshold for drug listing in NLEM year 2012. They recommended a ceiling threshold of cost-effective intervention at 160,000 THB per QALY gained [43,44]. In addition, we performed a fully incremental analysis to compare cost-effectiveness among all PCVs.

### 4.5. Sensitivity Analysis

We assessed how the results were changed when we altered inputs in our sensitivity analyses. We incorporated the herd effect in our sensitivity analysis for all population cohorts ranging from age 16 to 99 years. The herd effect of the vaccines against IPD was based on a previous meta-analysis [38], while the herd effect against pneumonia was calculated from the effect of PCV7 with a serotype coverage adjustment [39,45]. Because of the limited evidence of herd effect of PCV against pneumonia in Thailand, we derived the effect of PCV with serotype coverage adjustment using the following equation. We also performed analyses for a 3 + 1 dose of PCV.

%pneumonia reduction in Thailand = %hospitalized pneumonia reduction in the US × Serotype coverage in Thailand–Serotype coverage in the US

We varied the vaccine efficacy of PCV12 by 10–30% lower than full efficacy. The price of PCV12 was also varied from 50% to 150% of the proposed price (1.525 to 4.575 USD/dose). In addition, a probabilistic sensitivity analysis (PSA) was conducted to simultaneously examine the effects of all input uncertainty using a Monte Carlo simulation performed by Microsoft Excel 2003 (Microsoft Corp., Redmond, WA) [46]. A Monte Carlo simulation was run for 1000 sets of the simulation to give a range of values for total costs, outcomes, and ICERs. Results of the PSA were presented as a cost-effectiveness analysis plane and a cost-effectiveness acceptability curve. The expected net monetary benefit (NMB) was calculated for the WTP of the NLEM 2012 threshold in Thailand to show the probability that PCV12 is cost-effective for monetary values that a decision maker might be willing to pay.

### 4.6. Budget Impact Analysis

The budget impact analysis was performed to estimate the budget required for implementing a national PCV12 vaccination program compared to no vaccination among Thai infants in the year 2018–2022. The numbers of Thai infants from 2007 to 2017 were used to estimate the numbers in the years 2018–2022 using a linear trend relationship. The wastage rate of 1.1% was also applied based on a previous study in a Thai setting [42]. All cost values are presented in the year 2018, and readers can convert to US dollar ($) using the exchange rate THB 31.4 = $1 to compare across the country. The cost used for budget impact was not discounted. Because PCV12 vaccination could reduce future costs due to the prevention of pneumococcal infected diseases, cost offset was also considered to estimate the budget impact in addition to the whole required budget.

## 5. Results

### 5.1. Public Health Value of Vaccine Innovation

#### 5.1.1. Base-Case Analysis

The use of 2 + 1 PCV12 among a hypothetical cohort of 100,000 Thai infants was estimated to avert 5358 cases, including 5 pneumococcal meningitis, 43 pneumococcal bacteremia 5144 all-cause pneumonia, and 166 all-cause AOM compared to no vaccination. When compared to no vaccination, the implementation of a national 2 + 1 PCV12 immunization program was a cost-saving strategy. It could increase 0.0349 QALYs/person and reduce the total healthcare cost of 773 THB/person compared to no vaccination (Table 4). The incremental QALY gained for PCV12 and PCV13 compared to no vaccination was the same because we assumed the same vaccine efficacy for PCV12 and PCV13 as the prevalence of serotype 3 is very low among children, based on the serotype distribution in Thailand. On the other hand, implementing national 2 + 1 PCV10 and PCV13 increases both cost and QALYs compared to no vaccination. PCV10 would increase 0.0228 QALYs/person and 895 THB/person. Therefore, the ICER of PCV10 was 39,322 THB/QALY. Similarly, PCV13 would increase 0.0349 QALYs/person and 542 THB/person. Thus, the ICER of PCV13 was 15,523 THB/QALY (Table 4). Based on the Thai ceiling threshold of cost-effectiveness intervention (160,000 THB/QALY gained), both PCV10 and PCV13 were considered cost-effective interventions. A fully incremental analysis, comparing one PCV to another, revealed that PCV10 was dominated by PCV13. PCV13 could increase 0.0121 QALY/person and reduce total healthcare cost of 354 THB/person comparing to PCV10. However, PCV13 was also dominated by PCV12. PCV12 would produce QALY as same as PCV13, but it would reduce total healthcare cost by 1315 THB/person. Therefore, based on this estimation, PCV12 was the best option for Thai infants to prevent pneumococcal-related diseases (Table 5).

#### 5.1.2. Multivariate Probabilistic Sensitivity Analysis

**Figure 3:**
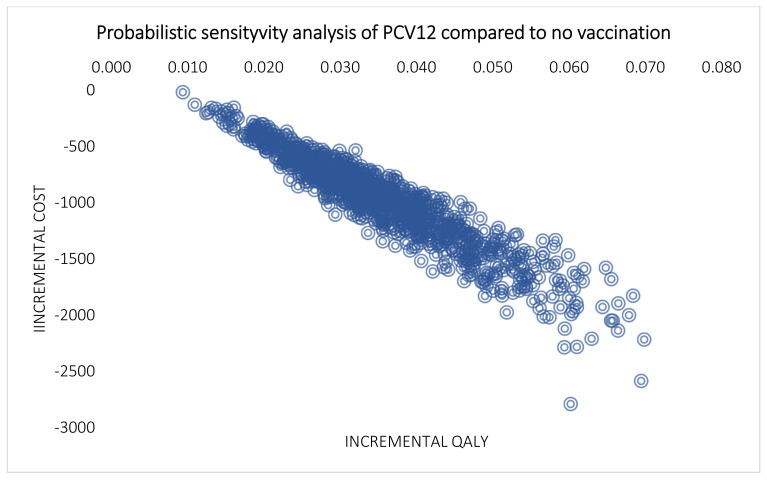
Multivariable probabilistic sensitivity analysis of PCV12 compared to no vaccination.

Results of the PSA are presented in the cost-effectiveness scatter plot in Figure 3. Despite variation in base-case inputs, all simulated incremental cost-effectiveness ratios were in the lower-right quadrant, indicating that implementing a national PCV12 immunization program was less costly and more effective than no vaccination (dominant). The cost-effectiveness acceptability curve indicated that the national PCV12 immunization program was a 100% probability of being cost-effective at all willingness-to-pay.

#### 5.1.3. Scenario Analyses

**Figure 4:**
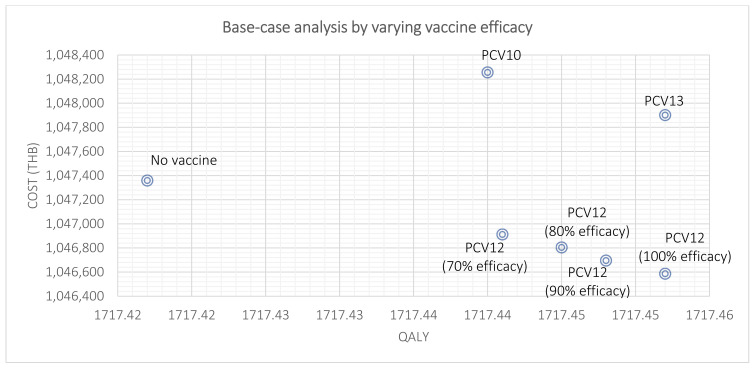
Scenario analysis by varying vaccine efficacy of PCV12 compared to other alternatives.

A scenario analysis by changing the PCV12 schedule from 2 + 1 to 3 + 1 indicated that PCV12 was still a cost-saving strategy. It reduced the total healthcare cost by 766 THB/person and increased 0.0380 QALYs compared to no vaccination. Scenario analyses by varying vaccine efficacy of 2 + 1 PCV12 by 10−30% lower than full efficacy indicated that PCV12 still reduced a total healthcare cost from 448 to 664 THB/person and increased QALYs from 0.0244 to 0.0314 QALYs (Table 6). The scenario analyses revealed that PCV12 was still a cost-saving strategy compared to no vaccination. However, PCV12 had a lower cost and QALY compared to PCV13 (Figure 4).

**Figure 5:**
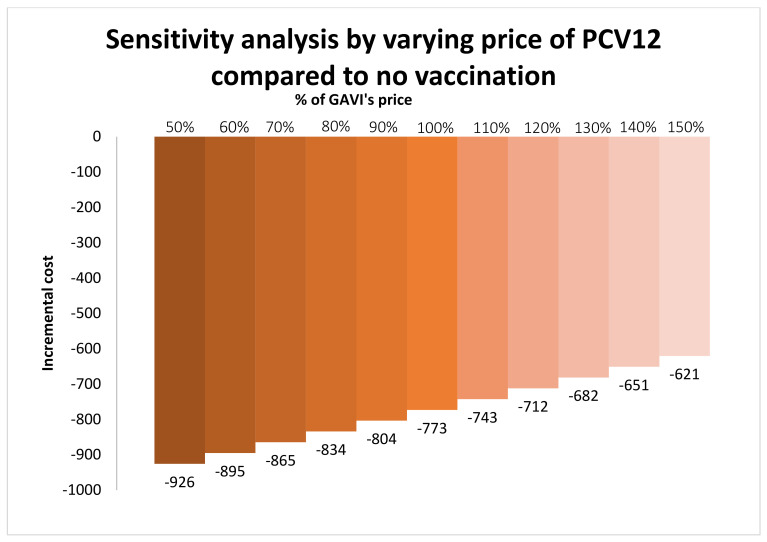
Scenario analysis by varying the price of PCV12 from 50% to 150% of the proposed price.

Another scenario analysis incorporating the herd-effect of PCV12 indicated that PCV12 reduced a total cost of 11,624 THB/person with an increase of 0.0897 QALY/person. The last scenario analysis varied the price of PCV12 from 50% to 150% of the proposed price (1.525 to 4.575 USD/dose). The analysis indicated that PCV12 could reduce a total healthcare cost from 621 to 926 THB/person. PCV12 did not increase the total healthcare cost at any price (Figure 5).

#### 5.1.4. Budget Impact Analysis

Budget impact analysis indicated that the Thai government should invest approximately 171–203 million baht to implement the national 2 + 1 PCV12 vaccination program for 2018–2022. However, when considering the cost offset from cases prevented by the 2 + 1 PCV12 vaccination program, the Thai government could save approximately 747–865 million baht at the same time (Table 7).

## 6. Discussion

### 6.1. Country Case Study–Thailand

The case study demonstrates that using PCV12 among Thai infants as part of the Expanded Programme on Immunization in Thailand would substantially reduce the incidence of IPD cases and death and lower total costs. The estimated budget implication for adopting PCV12 is around 200 million baht per year. Current evidence strongly supports the potential of using PCV12 for the national immunization program from an economic and investment perspective. The sensitivity analysis shows that the results are robust. When varying vaccine price or efficacy, PCV12 remains a cost-saving strategy.

Input parameters used in this model were contextualized for Thailand and obtained from high-quality sources, including systematic reviews and meta-analyses. The data on incidences, utility, and cost were used from local studies. Most updated vaccine efficacy and coverage data were used to reflect vaccine effectiveness that would apply to Thailand. With the use of more domestic inputs in the current analysis, it enables health authorities to be better informed in the decision-making process of introducing the PCV12 vaccine into the national immunization program.

Stakeholders felt optimistic about the findings and were excited regarding the opportunities for children in Thailand and other Gavi- and non-Gavi-eligible LMICs when PCV12 is available. The greatest concerns were the availability of limited information about upcoming PCV12 and decision-making uncertainties.

This study has some limitations. First, this study adopted static rather than dynamic transmission modeling. The static model used in the current analysis is unable to estimate the herd effect of vaccination. The model did not incorporate serotype replacement, which could lead to overestimating the benefits of PCV12 or implications for antimicrobial drug resistance. However, evidence of the indirect effect of PCV from various sources where PCV has been implemented was used. The indirect effect observed could be a combination of herd effect and serotype replacement. Second, the price assumption of PCV 12 was derived based on several assumptions, including the dynamic market of current PCV vaccines in Gavi eligible countries, the proposed PCV12 vaccine price by the manufacturer, and feedback from stakeholders meeting in Thailand. The actual price of PCV12 when launched might differ from the assumption used in the model and may affect the findings although scenario analyses showed the robustness of the findings. Third, we assumed the vaccine coverage of 100% based on the coverage rate of 96–99% for children’s vaccines listed in the Thai NIP. Although, we believe the coverage assumption is realistic based on the current vaccine coverage rate, the actual PCV12 coverage when introduced could be lower and the benefits may differ from our findings. Fourth, we assumed the benefit of herd immunity in individuals aged 16 and above was consistent with previous cost-effectiveness studies in Thailand. Therefore, the exclusion of herd effect in individuals younger than 16 years makes the vaccine benefits conservative and its inclusion may show better public health value. Last, we assumed the vaccine efficacy of PCV12 same as PCV13 because of the limited evidence of PCV12. The prevalence of serotype 3, which is not included in PCV12 but in PCV13, is very low, indicating the validity of the assumptions. However, the assumption might not hold true in the future and may alter the public health value of PCV12. We performed several sensitivity analyses varying the vaccine efficacy that demonstrated the robustness of the findings.

In summary, the use of PCV12 in the national immunization program for Thai children would be a highly cost-effective intervention and is likely to produce a budget impact affordable to the government. Given the above economic evidence, there is a high potential for PCV12 to provide value if it demonstrates the expected immunogenicity and safety upon completion of clinical trials and is registered and included in Thailand’s national vaccination program.

### 6.2. The Public Health Value of Vaccine Innovation

This investment case study for PCV 12, a vaccine under development, demonstrates the potential usefulness of this vaccine once approved. Economic analysis is an essential component of research during vaccine development to enable decisions and demonstrate public health value. In addition, vaccine developers need to understand the return on investment on the vaccines, from the company and country perspectives, to bring these to market, accounting for all development costs, benefits, and risks. Moreover, now that broader societal benefits of vaccines are increasingly recognized, accounting for such benefits should be considered in the future from the societal perspective.

## 7. Conclusions

In summary, an investment case study for a vaccine under development can be a valuable approach to understanding the public health value of the upcoming vaccine and exploring rational options for the introduction. Such analysis should explicitly state the limitations and uncertainty around the analysis so that decision makers are fully aware. In addition, understanding of return on investment for vaccine development and contextualizing broader benefits of vaccines can inform decision makers from a multi-stakeholder perspective.

## Figures and Tables

**Table 1:** Comparison of PCV in the current market.

Type	Adjuvant	Conjugation Method	Carrier Protein Content	Serotypes
PCV10 (Synflorix)	Alum	Bifunctional spacer	NTHi Protein D: 9–16 μg Tetanus toxoid: 5–10 μg Diphtheria toxoid: 3–6 μg	STs 1, 5, 6B, 7F, 9V, 14, 23F: 1 μg STs 4, 18C, 19F: 3 μg
PCV 13 (Prevnar 13)	Alum	Reductive animation	CRM_197_: 32 μg	ST 6B: 4.4 μg STs 1, 3, 4, 5, 6A, 7F, 9V, 14, 18C, 19A, 19F, 23F: 2.2 μg
PCV7 (Prevnar)	Alum	Reductive animation	CRM_197_: 20 μg	ST 6B: 4 μg STs 4, 9V, 14,18C, 19F, 23F: 2 μg

**Table 2:** SK-PCV12 Target Product Profile (TPP).

Variable	Target Product Profile (TPP)
Type	PCV 12
Serotypes	STs 1, 4, 5, 6A, 6B, 7F, 9V, 14, 18C, 19A, 19F and 23F conjugated to Diphtheria CRM197 protein (12 valent)
Product Registration Path	MFDS approval, followed by other NRAs WHO prequalification
The first NRA approval	2021
WHO Prequalification Date	2022
Target Populations	Children 6 weeks to 12 weeks of age
Target Countries	Mainly low-income countries (LIC), including GAVI countries
Primary Target Delivery Channel	Through GAVI or UNICEF delivery channels into the country, vaccination infrastructure
Efficacy Boundary and Primary Endpoint	Non-inferior immunogenicity compared to Prevnar 13 based on (1) the proportion of subjects with pneumococcal serotype-specific IgG antibody concentrations ≥ 0.35 μg/mL at 1-month post-dose 2, or (2) the pneumococcal serotype-specific IgG GMC at one-month post-dose 3
Co-administration	Can be safely co-administered with concomitantly scheduled licensed vaccines. Does not result in antigen interference with concomitantly scheduled licensed vaccines
Presentation	Two-dose vial
Formulation	Ready to use a liquid product
Doses	The 2 + 1-does immunization series at 6, 14 weeks, and 9–12 months of age
Administration	Intramuscular
Vaccine Volume (cm^3^/dose)	0.5 mL per dose
Stability	Must be stored at 2–8 °C using EPI-compatible cold chain systems Vaccine vial monitors are required to demonstrate that the cold chain has been maintained

Source: SK bioscience.

**Table 3:** Input parameters.

Parameter Description	Mean	SE	References
**Epidemiology parameters**			
**Annual incidence per 100,000 population for meningitis**	1.36	0.195	Meta-analysis [25,26,27]
**Annual incidence per 100,000 population for bacteremia**	11.10	8.102	[28]
**Annual incidence per 100,000 population for all cause pneumonia**	3627	570.73	[29]
**Annual incidence per 100,000 population for acute otitis media**	601.08	3.844	[30]
**Proportion of hospitalized pneumonia**	0.6741	N/A	[29]
**Epilepsy after pneumococcal meningitis**	0.0821286	0.0048154	NHSO
**Hearing loss after pneumococcal meningitis**	0.0163027	0.002221	NHSO
**Neurodevelopmental impairment after pneumococcal meningitis**	0.0018456	0.0007528	NHSO
**Hearing loss after AOM**	0.0065826	0.000957	NHSO
**Death after pneumococcal meningitis**	0.032258	0.031234	[31]
**Death after pneumococcal bacteremia**	0.080000	0.036693	[31]
**Death after hospitalized pneumonia**	0.014241	0.000408	[31]
**Vaccine efficacy (PCV7; 3 + 1 schedule)**			
**IPD caused by vaccine serotype**	89.00%	5.87%	[32]
**Clinical pneumonia**	25.50%	8.72%	[33]
**AOM**	6.00%	1.28%	[32]
**Vaccine efficacy (PCV10; 3 + 1 schedule)**			
**IPD is caused by vaccine serotype**	100.00%	0.18%	[34,35]
**Clinical pneumonia**	21.80%	6.63%	[35]
**AOM**	6.00%	1.28%	[32]
**Vaccine efficacy (PCV13; 3 + 1 schedule)**			
**IPD caused by vaccine serotype**	89.00%	5.87%	[32]
**Clinical pneumonia**	25.50%	8.72%	[33]
**AOM**	6.00%	1.28%	[32]
**Vaccine efficacy of PCV10; 2 + 1 schedule against IPD**	92.00%	10.71%	[34]
**Vaccine serotype coverage in Thai**			
**PCV7 serotype coverage in Thai <5**	74.11%	N/A	Meta-analysis [36,37]
**PCV7 serotype coverage in Thai 5–64**	47.82%	N/A	Meta-analysis [36,37]
**PCV7 serotype coverage in Thai ≥65**	48.11%	N/A	Meta-analysis [36,37]
**PCV10 serotype coverage in Thai <5**	78.72%	N/A	Meta-analysis [36,37]
**PCV10 serotype coverage in Thai 5–64**	54.73%	N/A	Meta-analysis [36,37]
**PCV10 serotype coverage in Thai ≥65**	55.30%	N/A	Meta-analysis [36,37]
**PCV13 serotype coverage in Thai <5**	91.73%	N/A	Meta-analysis [36,37]
**PCV13 serotype coverage in Thai 5–64**	76.04%	N/A	Meta-analysis [36,37]
**PCV13 serotype coverage in Thai ≥65**	77.60%	N/A	Meta-analysis [36,37]
**Vaccine serotype coverage US**			
**PCV7 serotype coverage in aged 10 to 39**	71.30%	N/A	[37]
**PCV7 serotype coverage in aged 40 to 64**	65.40%	N/A	[37]
**PCV7 serotype coverage in aged ≥65**	69.70%	N/A	[37]
**% Reduction in IPD (herd effects) from PCV7**			
**% Herd effects in aged 0–4**	38.00%	3.83%	[38]
**% Herd effects in aged 5–18**	19.00%	4.85%	[38]
**% Herd effects in aged 19–49**	15.00%	2.80%	[38]
**% Herd effects in aged 50–64**	22.00%	1.79%	[38]
**% Herd effects in aged ≥65**	23.00%	1.28%	[38]
**% Reduction in pneumonia from PCV7**			
**% Herd effects in aged 0–4**	43.2%	4.26%	[39]
**% Herd effects in aged 5–18**	4.50%	5.40%	[39]
**% Herd effects in aged 19–49**	7.80%	3.69%	[39]
**% Herd effects in aged 50–64**	0.00%	N/A	[39]
**% Herd effects in aged ≥65**	6.60%	3.11%	[39]
**% Reduction in IPD (herd effects) from PCV13**			
**% Herd effects in aged 0–4**	42.34%	N/A	[38]
**% Herd effects in aged 5–18**	36.82%	N/A	[38]
**% Herd effects in aged 19–49**	37.10%	N/A	[38]
**% Herd effects in aged 50–64**	37.10%	N/A	[38]
**% Herd effects in aged ≥65**	40.71%	N/A	[38]
**Cost parameters**			
**Vaccine cost**			
**PCV12 (USD/dose)**	3.05	-	[40]
**PCV10 (USD/dose)**	16.0	-	[40]
**PCV13 (USD/dose)**	16.2	-	[40]
**Direct medical cost**			
***Cost per episode***			
**Meningitis aged <=14**	88,863.7	5576.88	NHSO
**Meningitis aged 15 to 59**	83,063.84	4728.69	NHSO
**Meningitis aged >=60**	110,488.07	6313.6	NHSO
**Bacteremia aged <=14**	53,424.67	6526.55	NHSO
**Bacteremia aged 15 to 59**	65,466.53	4334.92	NHSO
**Bacteremia aged >=60**	76,565.08	3833.83	NHSO
**Hospitalized pneumonia aged <=14**	26,923.18	9099	NHSO
**Hospitalized pneumonia aged 15 to 59**	76,660.64	23,952	NHSO
**Hospitalized pneumonia aged >=60**	91,201.38	31,948	NHSO
**Non-hospitalized pneumonia aged <=14**	333.78	54.19	NHSO
**Non-hospitalized pneumonia aged 15 to 59**	771.58	169.09	NHSO
**Non-hospitalized pneumonia aged >=60**	640.76	84.81	NHSO
**AOM aged <=14**	379.33	4.53	NHSO
**AOM aged 15 to 59**	254.7	3.69	NHSO
**AOM aged >=60**	500.12	11.51	NHSO
***Cost per year***			
**Epilepsy aged <=14**	5589.81	64.15	NHSO
**Epilepsy aged 15 to 59**	7702.81	47.18	NHSO
**Epilepsy aged >=60**	14,288.80	127.57	NHSO
**Hearing loss aged <=14**	892.19	33.82	NHSO
**Hearing loss aged 15 to 59**	957.80	14.81	NHSO
**Hearing loss aged >=60**	877.37	9.93	NHSO
**Neurodevelopmental impairment aged <=14**	1796.98	37.23	NHSO
**Neurodevelopmental impairment aged 15 to 59**	4940.03	60.83	NHSO
**Neurodevelopmental impairment aged >=60**	1312.17	76.93	NHSO
**Chronic lung <=14**	1519	1404	[31]
**Chronic lung 15 to 59**	3576	62	[31]
**Chronic lung >=60**	3933	31	[31]
**Direct non-medical cost**			
**Direct non-medical cost for meningitis (per episode)**	18,121	N/A	Calculation
**Direct non-medical cost for bacteremia (per episode)**	11,687	N/A	Calculation
**Direct non-medical cost for pneumonia (per episode)**	5481	N/A	Calculation
**Direct non-medical cost for acute otitis media (per episode)**	617	N/A	Calculation
**Direct non-medical cost for epilepsy (per year)**	5253	N/A	Calculation
**Direct non-medical cost for hearing loss (per year)**	1015	N/A	Calculation
**Direct non-medical cost for neurodevelopmental impairment (per year)**	20,535	N/A	Calculation
**Direct non-medical cost for chronic lung disease (per year)**	8347	N/A	Calculation
**Utility**			
**Utility for meningitis**	0.9638	0.0046	[31]
**Utility for bacteremia**	0.9852	0.0025	[31]
**Utility for pneumonia**	0.9910	0.0020	[31]
**Utility for acute otitis media**	0.9984	0.0001	[31]
**Utility for epilepsy**	0.6400	0.0738	[31]
**Utility for hearing loss**	0.5500	0.0554	[31]
**Utility for neurodevelopmental impairment-mild mental retardation**	0.6900	0.0707	[31]
**Utility for neurodevelopmental impairment-severe mental retardation**	0.1000	0.1085	[31]
**Utility for neurodevelopmental impairment-mental retardation and epilepsy**	0.0001	0.0943	[31]
**Utility for chronic lung disease**	0.5900	0.0575	[31]

NHSO; National Health Security Office.

**Table 4:** Base-case analysis of PCV10, PCV12, and PCV13 compared to no vaccination.

Vaccine	Total Cost	LYs	QALYs	Incremental Cost (THB)	Incremental QALY	ICER/QALY
No vaccine	1,047,360	1,718.401	1,717.417	Reference	Reference	Reference
PCV10	1,048,256	1718.418	1717.440	895	0.0228	39,322
PCV12	1,046,587	1718.428	1717.452	−773	0.0349	Cost-saving
PCV13	1,047,902	1718.428	1717.452	542	0.0349	15,523

THB: Thai Baht, LYs: Life years, QALYs: Quality-adjusted life years, ICER: Incremental cost-effectiveness ratio/QALYs.

**Table 5:** Fully incremental analysis of PCV10, PCV12, PCV13 and no vaccination.

Vaccine	Total Cost	LYs	QALYs	Incremental Cost (THB)	Incremental QALY	ICER/QALY
No vaccine	1,047,360	1718.401	1717.417	Reference	Reference	Reference
PCV10	1,048,256	1718.418	1717.440	895	0.0228	Dominated by PCV13
PCV13	1,047,902	1718.428	1717.452	−354	0.0121	Dominated by PCV12
PCV12	1,046,587	1718.428	1717.452	−1315	0.0000	Cost-saving

THB: Thai Baht, LYs: Life years, QALYs: Quality-adjusted life years, ICER: Incremental cost-effectiveness ratio/QALYs.

**Table 6:** Scenario analyses by varying vaccine efficacy of PCV12 by 10–30% lower than full efficacy.

Scenario	Total Cost	QALYs	Incremental cost (THB)	Incremental QALY	ICER/QALY
No vaccine	1,047,360	1717.417	Reference	Reference	Reference
Full efficacy	1,046,587	1717.452	−773	0.0349	Cost-saving
10% reduction	1,046,696	1717.448	−664	0.0314	Cost-saving
20% reduction	1,046,805	1717.445	−556	0.0279	Cost-saving
30% reduction	1,046,913	1717.441	−448	0.0244	Cost-saving

THB: Thai Baht, QALYs: Quality adjusted life years, ICER: Incremental cost-effectiveness ratio/QALYs.

**Table 7:** Budget impact analysis from the years 2018–2022.

Budget Impact Analysis	Year				
	2018 (*n* = 668,470)	2019 (*n* = 653,624)	2020 (*n* = 631,355)	2021 (*n* = 631,355)	2022 (*n* = 631,355)
No consideration of cost offset	203 M	199 M	192 M	183 M	171 M
With consideration of cost offset *	−884 M	−864 M	−854 M	−796 M	−747 M

* negative value means saved cost, M: million Thai Baht, *n*: number of children estimated for the year.

## Data Availability

The data was available upon appropriate requests.
